# Subchronic Cannabidiol (CBD) Treatment During the Silent Period Fails to Prevent Increased Seizure Susceptibility Following Lithium-Pilocarpine-Induced Status Epilepticus

**DOI:** 10.3390/brainsci16080851

**Published:** 2026-08-11

**Authors:** Claudia Taborda Gómez, Florencia Fernández, Agustín Jara, Natalia Borda, Franco Moscovicz, Yessenia Yauri-Huaman, Rodrigo Caceres-Robles, Luis F. Pacheco-Otalora, Alberto Lazarowski, Jerónimo Auzmendi

**Affiliations:** 1 Instituto de Investigaciones Farmacológicas, Facultad de Farmacia y Bioquímica, Universidad de Buenos Aires, Ciudad Autónoma de Buenos Aires C1113AAA, Argentina; clamatago88@gmail.com (C.T.G.); fernandezfa91@gmail.com (F.F.); agustin_jara@icloud.com (A.J.);; 2 Laboratorio de Investigación en Neurociencias, Instituto Científico, Facultad de Ciencias de la Salud, Universidad Andina del Cusco, Cusco 08006, Peru; yessenia.yauri@unmsm.edu.pe (Y.Y.-H.); lpachecoo@uandina.edu.pe (L.F.P.-O.); 3 Instituto de Neurociencias de Castilla y León (INCYL), Universidad de Salamanca, 37007 Salamanca, Spain

**Keywords:** cannabidiol, seizure threshold, status epilepticus, epileptogenesis

## Abstract

**Highlights:**

**What are the main findings?**

- The susceptibility of rats to develop epileptic seizures after experiencing Status Epilepticus (seizure threshold) can be determined through the discrete administration of subconvulsive doses of pentylenetetrazol.
- Subchronic treatment (14 days) with Cannabidiol after Status Epilepticus does not modify the seizure threshold and increases several SE comorbidities such as the post-seizure irritability and weight loss.

**What are the implications of the main findings?**

- Based on the anti-inflammatory and anticonvulsant effects of Cannabidiol, it was hypothesized that subchronic treatment could improve the epileptic threshold after Status Epilepticus. Our results indicate that Cannabidiol, at least under the administered conditions, does not improve the epileptic threshold and exacerbate the post-SE comorbidities.

**Abstract:**

Introduction. In recent years, cannabidiol (CBD) has been used as an adjunct therapy to anti-seizure medications for the control of seizures in patients with drug-resistant epilepsy. In addition to its anticonvulsant effect, CBD also has well-defined anti-inflammatory properties. Since neuroinflammation can trigger various pro-epileptogenic mechanisms, CBD could play an inhibitory role in this process. Epileptogenesis is the process by which epilepsy becomes a chronic disease following a brain injury, and one of its main characteristics is an increased susceptibility to seizures due to a reduced seizure threshold. However, the role of CBD in modulating this susceptibility remains poorly understood. Methodology. We developed an experimental protocol to discretely measure the seizure threshold (DMST) 7 or 14 days after lithium-pilocarpine-induced status epilepticus (SE) through the administration of small intraperitoneal (i.p.) doses of pentylenetetrazol (15 mg/kg/every 10 min). Results. Using the DMST, we observed a significant decrease in the seizure threshold after SE associated with a hypersensitivity state characterized by irritability and marked weight loss. A separate cohort of rats was treated with CBD (20 mg/kg) for 14 days following SE. Conclusions. Treatment with CBD did not improve the seizure threshold; moreover, it worsened the hypersensitivity state and delayed recovery following SE. Our results suggest that orally administered CBD, at a human-equivalent therapeutic and safe dose, does not improve susceptibility to seizure development after SE.

## 1. Introduction

Cannabidiol (CBD), a phytocannabinoid without hallucinogenic effects, has been widely reported to have anticonvulsant and anti-inflammatory properties, highlighting its potential use in the treatment of drug-resistant epilepsy. Several clinical trials have shown that CBD as an adjuvant treatment can reduce the frequency and intensity of seizures in a wide range of patients with epileptic syndromes. These studies also demonstrated that CBD was well tolerated by most patients, with minimal adverse effects [1,2,3]. Based on this, the Food and Drug Administration approved its use as an adjunct to anti-seizure medication (ASM) treatment in Dravet and Lennox-Gastaut syndromes [4], although its mechanism of action has not been elucidated. Information from pharmacokinetic monitoring and ASM interactions shows that CBD treatment promotes an increase in ASM plasma concentrations [5], presumably by blocking cytochrome activity [6] and/or reducing the activity of ABC family transporters (ABC-t) [7,8,9]. CBD has been shown to interact with a variety of molecular targets, including cannabinoid, dopamine, serotonin, and opioid receptors, PPARγ, and ion channels such as TRPV1 and TRPV2 (Transient Receptor Potential Vanilloid 1 and 2 respectively) [10]. It has recently been suggested that CBD may enhance the effect of ASMs acting on the GABAergic system, while it may have a paradoxical effect when acting in combination with sodium channel blockers [11]. The complexity of interactions between CBD and its molecular targets suggests a multiplicity of effects that could regulate various cellular processes. In addition, several publications have demonstrated the anti-inflammatory potential of CBD and other cannabinoids through receptor activation, inhibition of the synthesis and release of inflammatory cytokines, inhibition of cell proliferation, and even induction of apoptosis [12,13,14]. Furthermore, it has been established that treatment with CBD decreases the cellular function of human neutrophils, monocytes, and eosinophils [15,16,17,18]. It has been suggested that neuroinflammation is a trigger for the development of neurodegenerative diseases [19]. Epileptogenesis is the process by which epilepsy develops into a chronic disease, increasing the susceptibility to seizure development [20]. It can be triggered by both genetic causes and acquired factors [20]. These factors include stroke, traumatic brain injury (TBI), and status epilepticus (SE), with different rates of epilepsy development in humans. Cases involving SE have a risk rate greater than 40% within the first few years after insult. Insults such as epileptic seizures without SE, stroke, and severe TBI are associated with a 10–20% risk of developing epilepsy, while mild TBI has a risk of less than 5% [21,22,23]. Despite its effectiveness in controlling seizures and its anti-inflammatory properties, it is unclear whether treatment with CBD can prevent epilepsy from becoming a chronic disease. Few experimental studies have addressed the role of CBD in epileptogenesis. When CBD was administered prior to the induction of SE by pilocarpine or during the development of kindling with pentylenetetrazol (PTZ), a decrease in the incidence of seizures was reported [24,25]. Similarly, chronic treatment with CBD during audiogenic kindling in susceptible rats prevented the recruitment of limbic structures [26]. However, all these results may be due to a decrease in the intensity of the insult rather than a direct action on the development of epileptogenesis. Using rats with fully kindled amygdala, partial seizure suppression was observed at high doses of CBD (>250 mg/kg) administered prior to testing [27]. On the other hand, using Scn1a +/− mice in which seizures are triggered by hyperthermia, subchronic treatment with CBD did not alter the frequency of spontaneous seizures [28]. Similar results were obtained in a model of cobalt-induced seizures [29]. By contrast, a recent article showed that chronic treatment with high doses of CBD after pilocarpine-induced SE produced a decrease in seizure burden; however, the experimental design included treatment with CBD prior to SE [30].

The aim of this study was to determine whether subchronic treatment with CBD can affect the susceptibility to seizure development. Given the variability in experimental approaches, routes of administration, doses, and duration of CBD treatment found in the literature, we used the lithium-pilocarpine model of SE induction. CBD was administered orally at the maximum daily dose recommended for clinical treatment (20 mg/kg) for 14 days, and susceptibility to seizure development was evaluated by the administration of subconvulsive PTZ.

## 2. Materials and Methods

### 2.1. Animal and Experimental Models

**Figure 1:**
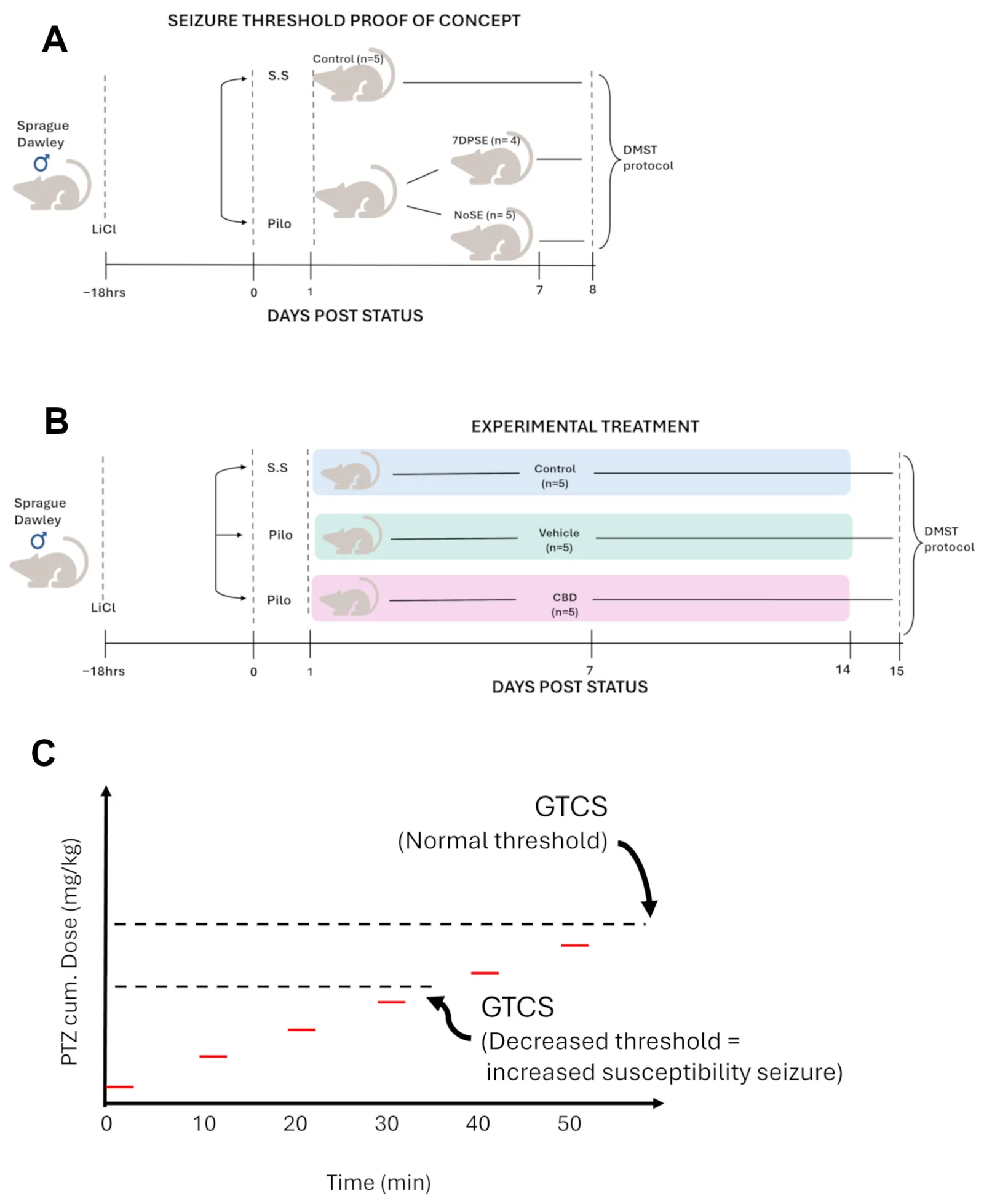
Experimental design and model development. (**A**) Schematic diagram of the experimental design for seizure threshold proof of concept showing the experimental groups as Control (treated with lithium chloride [LiCl] and saline solution [S.S]), 7DPSE (seven days post-SE, treated with lithium chloride [LiCl] and pilocarpine [Pilo]), and NoSE (same treatment as 7DPSE without development of SE). (**B**) The panel presents a schematic diagram of the experimental treatments. Control and convulsive groups were produced as above. All treatments were applied during 14 DPSE (14 days post-SE—highlighted areas) via gavage. Control (treated with water—light blue area), Vehicle (treated with sesame oil—light green area), and CBD (treated with CBD oil—pink area). In both experimental schemes, 24 h after the end of the experimental period, all groups were subjected to the DMST (discretely measured seizure threshold) protocol. (**C**) Diagram representing the DMST protocol. The red lines indicate the moment when subconvulsive doses of PTZ were administered. The dotted lines represent the threshold dose required to produce generalized tonic-colonic seizures (GTCS) in naive animals (normal threshold) and after SE (increased susceptibility threshold).

Male Sprague-Dawley rats weighing approximately 250 g at the start of the experiment were used. The animals were housed under standard conditions one week prior to the start of the experiment (12 h light/12 h dark, temperature 22 ± 2 °C, food and water ad libitum). Status epilepticus (SE) was induced by the lithium-pilocarpine paradigm as described previously [31]. Briefly, each animal was administered lithium chloride (127 mg/kg i.p.) since lithium potentiates and facilitates the induction of pilocarpine-induced epileptic seizures, reducing the pilocarpine dose by 10 times. Under these conditions, the use of a muscarinic antagonist, such as atropine or methylatropine, is not considered essential, as the peripheral effects of pilocarpine are substantially reduced. After 18 h, pilocarpine (30 mg/kg i.p.) was administered to induce SE, and convulsive behavior was assessed using the Racine scale (Figure 1). The control group was treated with a similar volume of saline solution i.p. instead of pilocarpine. SE was considered achieved when the animals presented generalized tonic-clonic seizures (GTCS) for at least 5 min. Thirty minutes after the onset of SE, diazepam (DZP, 50 mg/kg i.p.) was administered. SE was considered terminated when the seizures ceased. Following SE, the rats were housed individually to prevent injuries resulting from postictal aggressive behavior. Animals remained individually housed throughout the entire treatment period under standard conditions. The animals were monitored for a period of 48 h, during which their vital signs were assessed to ensure recovery from SE. Animals exhibiting prolonged respiratory distress, weight loss exceeding 50% of their initial body weight, severe dehydration, or visible signs of necrosis (cyanotic discoloration) on the tail or hind limbs were euthanized in accordance with resolution REDEC-2025-2661-E-UBA-DCT_FFYB of the Institutional Committee for the Care and Use of Laboratory Animals of the Faculty of Pharmacy and Biochemistry, University of Buenos Aires. The control group was treated with lithium and an equivalent volume of saline solution, followed one hour later by a single dose of DZP.

### 2.2. Experimental Design

In this study, we used 31 rats, 10 of which formed the control group, while the remaining rats were subjected to the lithium-pilocarpine paradigm. Only 14 rats developed SE and were divided into three experimental groups. Four SE rats and five control rats were reserved for the epileptic threshold proof-of-concept test (Figure 1A) at 7 days post-SE (7 DPSE). The other groups, meanwhile, were treated orally for 14 days post-SE (14 DPSE) (Figure 1B). The control group (*n* = 5) was administered an equivalent volume of water. The CBD group (*n* = 5) was treated with a dose of 20 mg/kg CBD every 24 h (Kanbis^®^, 99% pure cannabidiol, CBD 100 mg/mL in sesame oil, Elea, Buenos Aires, Argentina). Finally, the vehicle group (*n* = 5) was treated with a proportional volume of sesame oil every 24 h. All treatments were administered via gavage after recording animal weights and handling indices.

### 2.3. Evaluation of Discretely Measured Seizure Threshold (DMST)

At 24 h after the end of the experimental period for each group (7 or 14 DPSE), the seizure threshold was evaluated using the DMST protocol adapted from the rapid kindling protocol [31]. PTZ is rapidly absorbed (1–3 min i.p.), with a half-life of 116 ± 25 min, a clearance of 5.36 ± 0.34 mL/min/kg, and very low protein binding [32]. In addition, PTZ typically exhibits a sigmoidal dose–response relationship, with subthreshold doses in the range of <20–30 mg/kg [32]. Finally, the DMST protocol consists of the administration of successive subconvulsive doses (15 mg/kg) of pentylenetetrazol (PTZ) intraperitoneally every 10 min. During the interval between doses, the animals were observed, and the seizure threshold was established when they presented with GTCS (Figure 1C). For each animal, the cumulative dose and latency to GTCS were recorded.

### 2.4. Monitoring Animal Development

*A. Weight evolution.* The body weight of each animal was recorded daily. The weight measured on the day of SE induction was established as the reference value (100%), and the values for consecutive days were expressed as a percentage of that baseline weight. Based on these data, a sixth-degree polynomial was fitted to describe the temporal fluctuations in weight. The parameters defining the growth model of the animals after SE were defined as $T_{min}$: (time at which maximum weight loss occurred); $T_{rec}$ (time at which the animals reached 100% of their initial weight); $T_{max}$ (time at which the animals reached their maximum weight); ${Weight}_{loss}$ (difference between initial and minimum weight); and ${Weight}_{gain}$ (difference between maximum and initial weight). The parameters for each animal were obtained from the models and then averaged, and the adjusted average growth curve was plotted with its confidence interval.

*B. Manipulation Score:* The reaction to daily handling was assessed using a behavioral scale designed based on a previous handling score [33,34], adapted to our observations of the animals following exposure to the Li-Pilo paradigm. Each animal was assigned a daily score from 1 to 10, depending on its behavior during the weighing procedure. Reactivity to handling was always recorded at the same time and under the same conditions. The levels of the scale were defined as follows:

1: Normal, active, and docile in the cage before and during handling.
2: Active and alert, but elusive, responds well to handling.
3: Decreased activity but alert, responds to handling.
4: Lethargic, unresponsive to handling.
5: Lethargic, responsive to handling.
6: Slight difficulty handling inside the cage (exaggerated response to minor noises, difficult to remove from cage).
7: Moderate difficulty handling inside the cage (exaggerated response to minor noises, jumps uncontrollably, difficult to remove from cage).
8: Moderate difficulty handling outside the cage (flight response, jumps uncontrollably).
9: Severe difficulty handling inside and outside the cage (jumps uncontrollably, spins around on its tail).
10: Maximum difficulty handling (jumps uncontrollably, spins around on its tail, bites).

### 2.5. Statistical Analysis

All statistical tests were performed using GraphPad Prism 8.0.2. Parameters obtained from the weight evolution fitting and seizure threshold (control vs. 7DPSE and 7DPSE vs. 14DPSE) were compared using a two-tailed Student’s *t*-test with a significance level of 0.05. Correlation analyses were made via two-tailed linear regression with the same significance level. The seizure threshold to explore the effects of CBD was conducted by one-way ANOVA following Tukey’s multiple comparisons post-test with a significant level of 0.05. The behavioral analysis was carried out via two-way ANOVA, where the differences between each treatment and their interactions were evaluated using a *p*-value of 0.05.

## 3. Results

**Figure 2:**
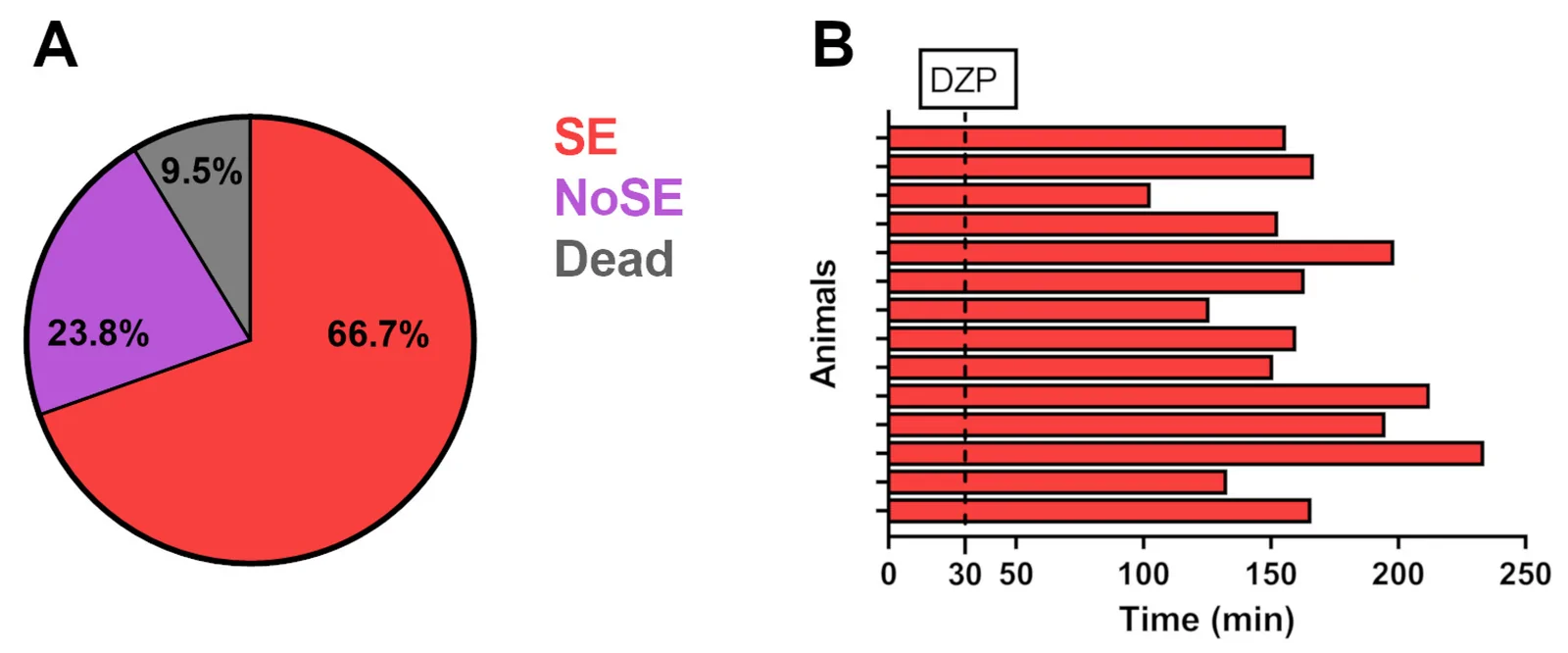
Development of lithium-pilocarpine model. (**A**) Pie chart showing the evolution of animals treated with lithium-pilocarpine (*n* = 21). (**B**) Individual responses of animals that developed SE. Each bar represents the time at which the animals stopped showing seizures as a result of the administration of a dose of diazepam (dotted line).

To begin investigating whether CBD treatment contributes to mitigating the susceptibility to seizures after SE, out of a total of 31 male rats, 21 rats were subjected to lithium-pilocarpine-induced SE, while the remaining 10 rats comprised the control group (Figure 1A). Of the total number of rats subjected to SE, 16 developed SE and 5 showed tonic-clonic seizures without developing SE (Figure 1A, NoSE group). The SE mortality rate was 12.5% (2/16 rats that developed SE—Figure 2A). Thirty minutes after the onset of SE, rats received a single dose of DZP, and the time until the animals stopped showing convulsive activity was recorded. Figure 2B shows the individual seizure duration for each animal that survived to SE induction (14/16), which ranged from 103 to 234 min, with a mean of 171.2 ± 36.02 min and a mean coefficient of variation of 21.04%.

**Figure 3:**
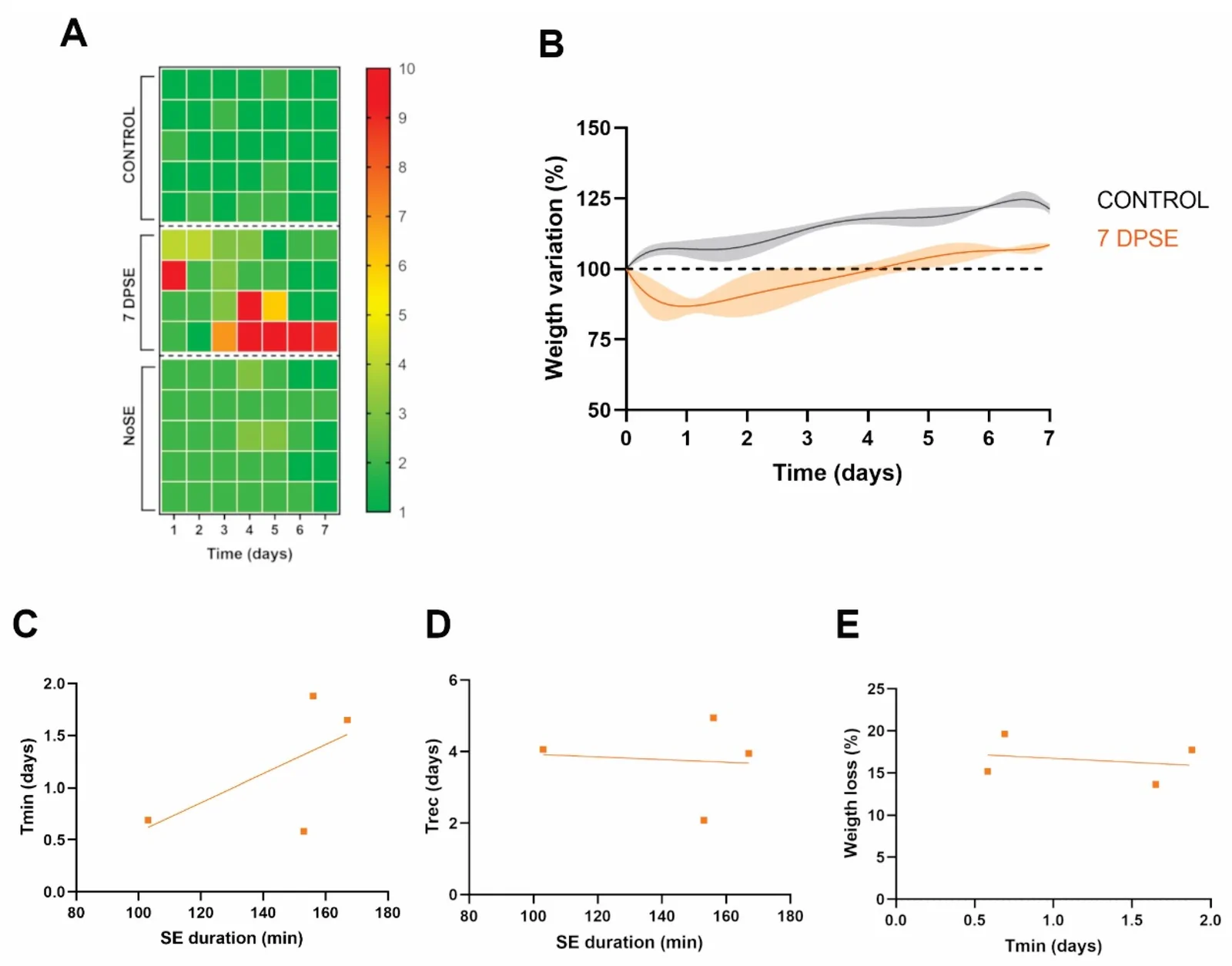
Evolution of rats’ hypersensitivity state post-SE. (**A**) Individual manipulation score. (**B**) Average evolution of animal weight obtained from individual polynomial fitting (dark line) plotted with the confidence interval (light line). (**C**–**E**) Correlation between SE duration and weight evolution parameters [$T_{min}$: (time at which maximum weight loss occurred); $T_{rec}$ (time at which the animals reached 100% of their initial weight); ${Weight}_{loss}$ (difference between initial and minimum weight)]. Non-significant correlations were observed (*p* = 0.390; *p* = 0.4558; *p* = 0.3827; respectively).

Take into account that aggressive behavior reported after SE, we recorded the rats’ responses to daily manipulation during the postictal period and the beginning of the silent period (7 days after SE induction). To describe this behavior, it was necessary to design an ad hoc scale (manipulation score) that described behaviors ranging from normal to lethargic and were exacerbated as a result of SE. Figure 3A shows the individual manipulation score for each rat in the control, 7DPSE, and NoSE groups. Rats that developed SE were more reactive to manipulation than the control group (*p* = 0.005). We observed a heterogeneous response in the SE group, as some rats showed high manipulation scores in the first few days after SE, while another subgroup of animals showed exacerbated responses from the third day after SE (pscore = 0.008; ptime > 0.05; interaction = ns). On the other hand, the animals in the NoSE group showed manipulation scores as low as those in the control group, suggesting that reactive behavior could be related to the intensity of the seizures rather than the time elapsed since SE induction.

The effect of SE was also reflected in weight evolution, showing on average ${Weight}_{loss}$ = 16.5 ± 2.7% from their initial weight, with a time elapsed to reach the minimum weight $T_{min}$ = 1.19 ± 0.65 days (Figure 3B). The time to recover that initial weight varied between 2.08 and 4.94 days ($T_{rec}$ = 3.75 ± 1.12 days), and subsequent weight gain ranged between 7.6 and 10.4% (${Weight}_{gain}$ = 9.3 ± 1.2%). This behavior differed from the control group, which showed continuous growth during the same period. 

Considering the variability in both the duration of SE and the parameters derived from the evolution of the animals’ weight, we examined whether there was a correlation between seizure duration and the animals’ response after SE. We did not observe a significant correlation between the SE duration and the assessed parameters (Figure 3C–E), suggesting that each response to SE was individual, even though they showed similar profiles.

### 3.1. Seizure Threshold Measure

**Figure 4:**
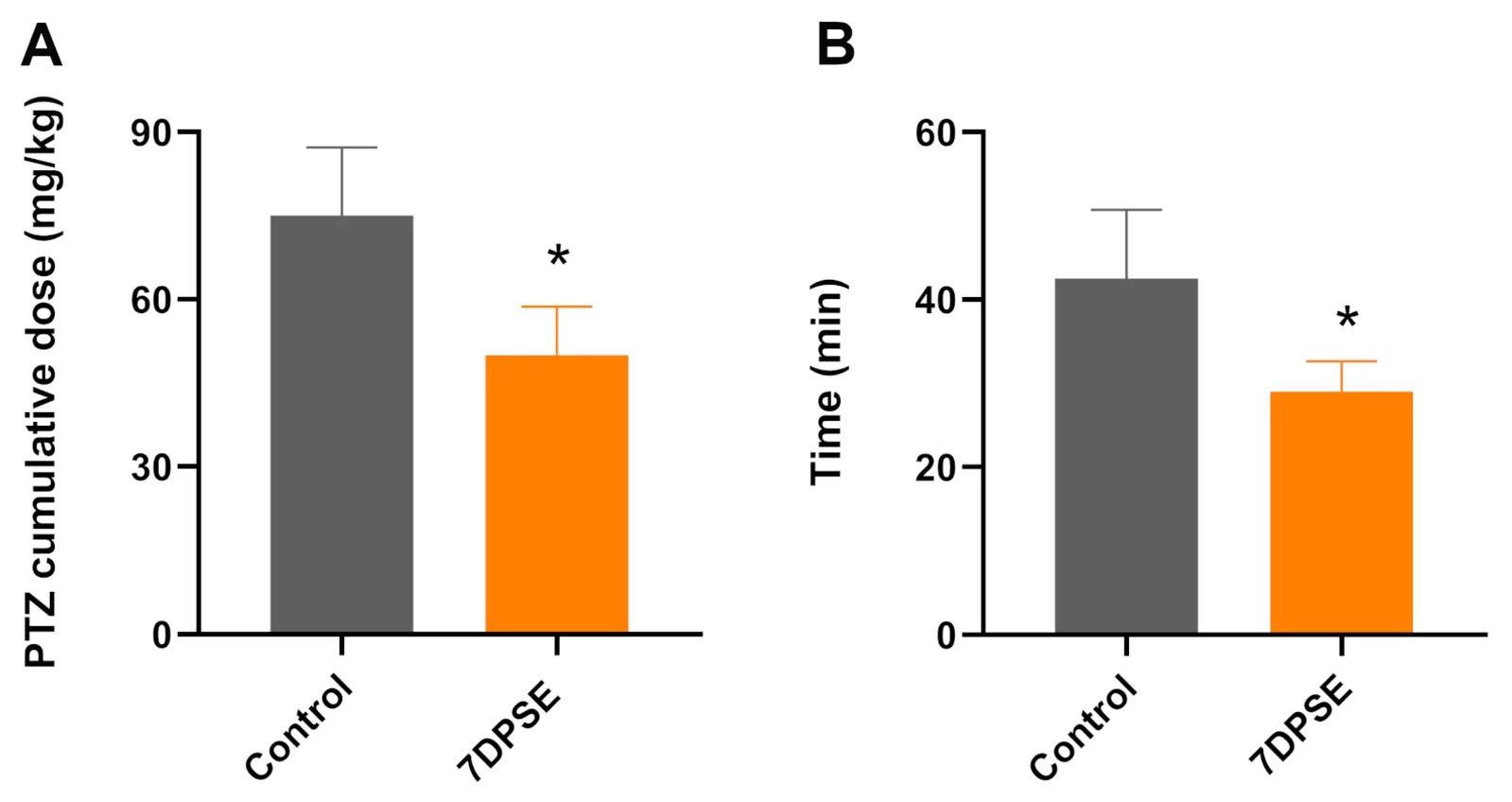
Evaluation of the seizure threshold. (**A**) Comparative cumulative dose of PTZ required to produce GTCS between Control group and 7DPSE (7 days post-SE). (**B**) Latency to the onset of GTCS. Data are shown as mean ± SD. Differences were analyzed using a Student’s *t*-test (* *p* < 0.05).

Our next step was to confirm whether the susceptibility to seizures increased as a result of SE. To do this, we designed a DMST protocol in which we administered subconvulsive doses of PTZ every ten minutes until the rats developed GTCS (Figure 1C). The animals that suffered SE required 30% less PTZ dosage to generate GTCS (Control: 75 ± 12.25 mg/kg vs. 7DPSE: 50 ± 8.6 mg/kg, Figure 4A). Figure 4B shows a similar change in the latency for the development of GTCS from 42.5 ± 8.2 min vs. 29 ± 3.6 min. Taken together, these results suggest that SE increases the susceptibility to developing seizures 7 days after SE.

### 3.2. Evaluation of the Effect of CBD on Susceptibility to Develop Seizures After SE

**Figure 5:**
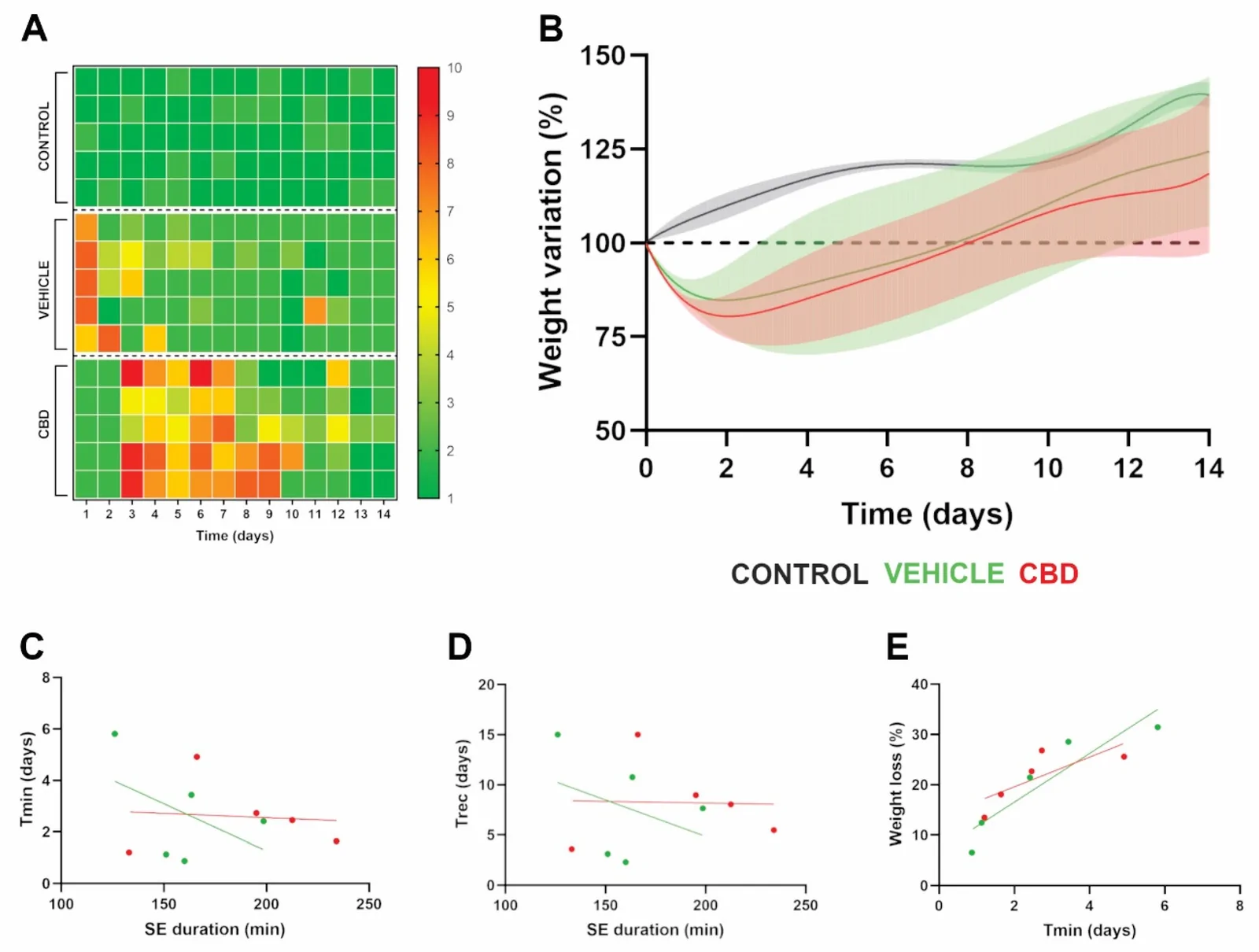
CBD treatment effect. (**A**) Individual manipulation score. (**B**) Average evolution of animal weights obtained from individual polynomial fitting (dark line) plotted with the confidence interval (light line). (**C**–**E**) Correlation between SE duration and weight evolution parameters [$T_{min}$: (time at which maximum weight loss occurred); $T_{rec}$ (time at which the animals reached 100% of their initial weight); ${Weight}_{loss}$ (difference between initial and minimum weight)]. Non-significant correlations were observed for both treatments ((**C**) *p*= 0.8833 to CBD; *p* = 0.4165 to vehicle and (**D**) *p* = 0.9619 to CBD; *p* = 0.5536 to vehicle). A positive correlation between $T_{min}$ and ${Weight}_{loss}$ was observed for both treatments ((**E**) *p* = 0.01296, r = 2.96 for CBD and *p* = 0.0260, r = 4.84 for vehicle). (**B**–**E**) Vehicle and CBD treatments are plotted in green and red, respectively.

Since CBD has well-documented anticonvulsant and anti-inflammatory effects, we evaluated whether treatment with CBD can modify the susceptibility to seizure development after SE. To do this, animals that developed SE were divided into two groups and treated with CBD or vehicle for 14 days, and the susceptibility to seizure development was evaluated using the DMST protocol (Figure 1). We studied individual behavior during treatment using the manipulation score. Figure 5A shows the behavioral evolution of the control group and the groups treated with vehicle or CBD. The control group maintained normal behavior throughout the experimental period, with scores between 1 and 2 on the scale, as they did not develop SE. As expected, rats treated with vehicle showed a period of decreasing reactivity during the 2–3 days post-SE, although we did not observe the same heterogeneous behavior seen at 7 DPSE. Strikingly, the group treated with CBD showed homogeneous and atypical behavior. During the first two days, the animals remained as unresponsive as the control group; however, from day 3 onward, the animals exhibited a markedly increased reactivity that persisted for a longer period than that of the group treated with vehicle (pscore < 0.0001; ptime = 0.0002; interaction < 0.0001). Despite these noticeable changes in the rats’ behavior, weight changes did not show significant differences between the vehicle and CBD treatments, as shown in Figure 5B. As observed in the analysis of parameters after SE, the growth parameters obtained for each treatments did not show a correlation with the duration of SE (Figure 5C,D). Conversely, ${Weight}_{loss}$ positively correlated with $T_{min}$ for both the vehicle and CBD treatments (*p* = 0.026 and *p* = 0.0129 respectively).

**Figure 6:**
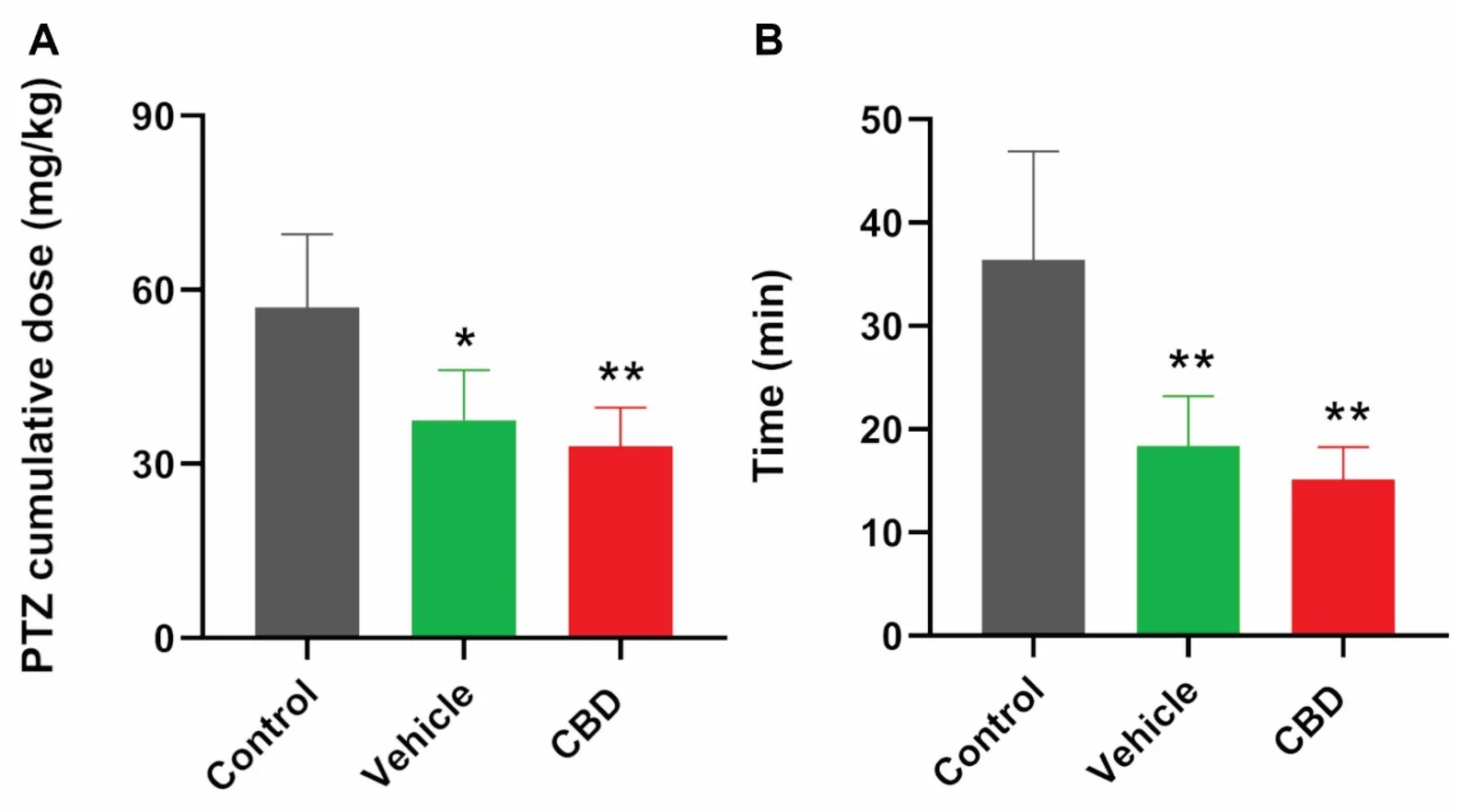
CBD treatment fails to improve seizure susceptibility after SE. (**A**) Accumulative doses of PTZ needed to trigger GTCS. (**B**) Latency to the onset of GCTS. Results are presented as mean ± SD. Differences were analyzed using one-way ANOVA following by Tukey’s multiple comparisons post-test (* *p* < 0.05; ** *p* < 0.01).

Given that the animals showed a distinctive behavioral profile for each treatment, we evaluated whether treatment with CBD could produce a shift in the growth parameters established after SE. Given the time difference between treatments (7 vs. 14 DPSE), we only evaluated $T_{min}$, $T_{rec}$, and ${Weight}_{loss}$. We observed that CBD treatment produced an increase in $T_{min}$ (1.61 ± 0.57 days vs. 2.59 ± 1.43 days; *p* = 0.036) and $T_{rec}$ (3.76 ± 1.20 days vs. 8.02 ± 3.96 days; *p* = 0.039) and a tendency to increase ${Weight}_{loss}$ (16.54 ± 2.66% vs. 21.34 ± 5.53%; *p* = 0.079). Taken together, these results suggest that CBD modifies the parameters established for SE and may have no effect on seizure susceptibility. For this reason, we evaluated the seizure threshold after each treatment using the DMST. Figure 6A shows a decrease in the PTZ doses required for the development of GTCS for both the vehicle and CBD treatments. This was also reflected in the latency to GTCS (Figure 6B), suggesting that CBD treatment does not affect the susceptibility to seizure development after pilocarpine-induced SE.

## 4. Discussion

In this study, we evaluated whether CBD administered orally at a human-equivalent maximum therapeutic daily dose (20 mg/kg) can prevent epilepsy from becoming a chronic disease. In this context, few studies have experimentally addressed this issue, and certain experimental approaches may lead to misinterpretation of the results, confusing anticonvulsant activity with anti-epileptogenic potential.

Several mechanisms, such as loss of GABAergic neurons, abnormal axon growth, changes in the number of synaptic terminals, and changes in the composition and/or gene variants in ion channels, have been proposed to explain the process by which epilepsy becomes a chronic disease after brain injury [25,35,36,37,38]. However, none of these mechanisms has been entirely conclusive. In this regard, processes such as hypoxia, oxidative stress, and neuroinflammation have been described as key interconnected factors in the development of neurodegenerative diseases [19]. This is where CBD could play an important role, as it has been described as having anticonvulsant and anti-inflammatory activities [12,13,14].

Considering that SE is the most prevalent cause of epilepsy becoming a chronic disease [20], we induced SE using the lithium-pilocarpine model, and we measured the animals’ response post-SE. We observed a state of hypersensitivity represented by an increase in aggressive behavior, as described in the literature on epilepsy animal models and patients [39,40]. These behavioral changes were accompanied by a marked decrease in weight (Figure 3), as we previously reported [31]. Strikingly, rats that did not develop SE showed a similar behavior as the control group even though they experienced GTCS, suggesting that the intensity and/or duration of the seizures affects this hypersensitivity state. This is directly in line with previously reported results, which showed a positive correlation between weight loss and the duration of SE [31]. Given that prolonged SE is associated with a high mortality rate, we administered DZP 30 min after the onset of SE to improve animal survival. However, the animals exhibited spontaneous recurrent seizures (SRS) over a highly variable time frame [41,42]; therefore, we developed the DMST to assess the susceptibility to seizures without having to wait for the onset of SRS. Ideally, seizures could be monitored using EEG recordings. The lack of such recordings could represent a significant limitation in our study; however, measuring the seizure threshold in conjunction with behavioral observations may provide a good approximation. Compared to the traditional method of measuring the epileptic threshold, which involves administering a continuous flow of PTZ through the rat’s tail vein, the DMST does not use PTZ administration via low-flow peristaltic pumps. Infusion rates as low as 0.1–0.5 mL/min are difficult to maintain. Furthermore, direct infusion can interfere with the animal’s free movement, causing mechanical problems with the infusion flow, damage to the catheter by the animal, and even disconnection of the line.

On the other hand, although the DMST may not show such a direct relationship between the PTZ dose and seizure activity, the published pharmacokinetic data for PTZ suggest that this issue will be minimal, since PTZ absorption via the intraperitoneal route is rapid (1–3 min) and its half-life is approximately 2 h [32]. Furthermore, to minimize this effect, we considered the onset of GTCS as the cutoff point for determining the epileptic threshold. Given the experimental design of the DMST, both the time required to determine the seizure threshold (approximately one hour versus a few minutes) and repeated handling of the animals could affect the determination of the threshold. In a seminal study conducted in our laboratory, we tested these variables and found that they did not affect the determination of the threshold in either normal mice or mice susceptible to seizures [43]. Using the DMST, we showed a greater susceptibility to developing seizures in 7DPSE rats, reflected by a lower PTZ dose and a decrease in the latency to develop GTCS. These results are consistent with those reported by other researchers using continuous measurement of the seizure threshold, who observed a decrease in the seizure threshold within 1 day to 6 months after the SE [44,45,46].

An additional limitation of the DMST protocol is the lack of a positive control to validate the detection of anti-epileptogenic effects. To date, none of the conventional anti-seizure medications have consistently demonstrated disease-modifying or anti-epileptogenic efficacy when administered after status epilepticus. In this context, rapamycin could represent a suitable positive control, as it has been shown to attenuate aberrant mossy fiber sprouting through inhibition of the mTOR signaling pathway. However, the reported anti-epileptogenic effects of rapamycin are variable and depend on the experimental model, timing of administration, and treatment paradigm [47,48]. Therefore, rapamycin cannot yet be considered a universally accepted positive control for studies on epileptogenesis. The absence of a universally accepted anti-epileptogenic positive control highlights the need for translational studies evaluating clinically relevant therapeutic strategies, such as post-status epilepticus cannabidiol administration.

Our next step was to investigate whether oral treatment with CBD oil after SE induction could improve the seizure threshold. This experimental scheme was used to prevent the anticonvulsant action of CBD from interfering with the injury caused by SE. It should be noted that experiments in which CBD has been administered before or during brain injury have pointed to a potential anti-epileptogenic effect of CBD [24,26,30,49,50]. However, these experimental designs, in addition to increasing the probability of false positives, are also far removed from clinical situations where CBD could be applied, i.e., once brain injury has already occurred. Surprisingly, we did not observe an increase in the seizure threshold after administering CBD for 14 days at a daily dose of 20 mg/kg. Also, aggressive behavior and weight loss were sustained. Strikingly, treatment with CBD deepened irritability and prolonged post-SE recovery times ($T_{min}$ and $T_{rec}$) (Figure 4), suggesting that CBD treatment could contribute to a decrease in the seizure threshold. This phenomenon may be explained by several, non-mutually exclusive mechanisms. First, CBD may interfere with the synaptic plasticity required for functional recovery following status epilepticus and/or suppress the microglial response necessary for the clearance of damaged cells and tissue remodeling [51,52,53]. Second, status epilepticus induces profound alterations in the expression and function of several molecular targets involved in the anxiolytic actions of CBD, including 5-HT1A serotonin receptors, TRPV1 channels, GPR55, and components of the endocannabinoid system [51,52,54,55]. Furthermore, one might assume that the observed weight loss is due to a disruption in food intake caused by the oily composition of the CBD carrier (sesame oil). However, weight loss is a characteristic of SE that was also observed in 7DPSE animals that did not receive additional treatment following SE. Nevertheless, our findings may be limited by the CBD administration regimen used, as its pharmacokinetics differ between humans and rodents. While the bioavailability of orally administered CBD is low in humans, it is typically higher in rodents due to faster absorption (0.5–2 h in rodents vs. 1–4 h in humans), although these values are comparable to those observed with a high-fat diet [56,57,58]. On the other hand, the half-life of a single oral dose of CBD oil is shorter in rodents (2–6 h in rodents vs. 1–10 h in humans) [57,59]. These differences may be even more pronounced in subchronic treatment, where the half-life in rats can be as long as 12 h, while in humans it ranges from 2 to 5 days [56,59], because rodents have a much faster metabolism and the accumulation of CBD in fat has less of an impact than it does in humans. Therefore, it is expected that a regimen using more daily applications and/or higher doses will yield different results. Under such dosing regimens, the difference between peak (C_max_) and trough (C_min_) plasma concentrations would be substantially reduced, thereby providing a more stable systemic exposure to CBD. Nevertheless, our dosing schedule was chosen considering that excessive animal handling, particularly during the early post-SE period, may increase stress and hyperexcitability, potentially triggering seizures and influencing the seizure threshold. In addition, the metabolic burden associated with recovery from SE could be further aggravated by gastrointestinal adverse effects associated with higher CBD doses. In this regard, Colasanti and colleagues administered two daily doses of 60 mg/kg of CBD following brain injury and found no changes in the seizure threshold [29]. Similarly, Anderson and colleagues demonstrated that doses of 100 mg/kg of CBD, as well as doses of 0.1 mg/kg of THC—either alone or in combination—had an anticonvulsant effect on hyperthermia-induced seizures in mice, whereas chronic treatment with doses of 500–1000 mg/kg/day of CBD did not alter the frequency of SRS. Even the co-administration of 130 mg/kg/day of CBD + 5 mg/kg/day of THC administered orally increased seizure severity and the mortality rate [28].

Since CBD inhibits the cytochromes CYP 3A4 and CYP 2C19 [60], subchronic treatment could lead to drug–drug interactions with PTZ, altering the DMST results and producing false-negative results. For this reason, the DMST measurement was performed 24 h after the last administration of CBD, which corresponds to 2–4 half-lives of CBD. In this context, the results obtained using the DMST protocol were statistically indistinguishable between the vehicle and CBD treatments (Figure 6) and showed a level similar to those obtained with the 7DPSE rats.

It is known that CBD has an inverted U-shaped dose–response curve for its effects on anxiety, sleep disturbance, substance use disorder, and arthritis, among other conditions [61,62,63,64]. However, regarding seizures, CBD presents a traditional sigmoidal curve [65,66], and it could be expected that its effect on the susceptibility to seizure development would have a similar profile. A recent meta-analysis found that prolonged use of CBD for seizure control was associated with a higher risk of adverse effects compared to the placebo and emphasized the importance of adjusting doses to avoid them. For these reasons, acute treatment with high doses of CBD could be effective in reducing the susceptibility to seizures. However, high concentrations of CBD (at µM range) have been shown to have a cytotoxic effect [16,67,68,69]; therefore, determining the therapeutic window for the prophylactic treatment of epileptogenesis after brain injury is key to working with high concentrations of CBD. Another factor that could influence our results is the route of administration. A recent article showed that inhaled CBD is much more potent than the oral route for controlling kainic acid-induced seizures, reducing the expression of IL-6, IL-33, and BDNF both in the brain and peripherally [70]. In this regard, absorption via inhalation is much faster than oral administration (5 min vs. 0.5–2 h), while the peak concentration is 4–6 times higher with the inhalation route.

Another limitation of the present study is that CBD concentrations were not determined in brain parenchyma after the 14-day treatment period. Several studies have demonstrated that, following oral administration, CBD concentrations in the brain exhibit a pharmacokinetic profile similar to that observed in plasma, with peak levels occurring shortly after dosing followed by a progressive decline over time [56,71]. Although the selected dosing regimen was based on its clinical relevance, brain drug concentrations provide a more direct measure of CNS exposure than plasma levels and are essential for determining whether therapeutically relevant tissue concentrations are maintained throughout treatment. Therefore, we cannot exclude the possibility that limited or fluctuating brain exposure contributed to the lack of anti-epileptogenic efficacy observed under our experimental conditions.

Although the present study did not demonstrate an anti-epileptogenic effect of CBD administered as a monotherapy after status epilepticus, these findings do not necessarily exclude a potential therapeutic role for CBD as an adjunctive treatment. Clinical and experimental studies have shown that CBD can enhance the anti-seizure efficacy of several anti-seizure medications, including clobazam, valproate, and levetiracetam, through both pharmacokinetic and pharmacodynamic interactions [72,73,74]. However, current evidence mainly supports an anticonvulsant rather than a disease-modifying effect. Whether subchronic treatment with CBD after SE can modify epileptogenic mechanisms in a manner that subsequently enhances the efficacy of anti-seizure medications remains unknown.

## 5. Conclusions

In summary, our findings suggest that the subchronic oral administration of CBD at 20 mg/kg does not reduce the susceptibility to seizure development following status epilepticus under the experimental conditions employed in this study. Further studies exploring different administration routes, alternative dosing regimens, and chronic treatment protocols, as well as monitoring spontaneous recurrent seizures, are necessary to definitively determine whether CBD possesses a true antiepileptogenic effect.

## Data Availability

For privacy reasons, all data supporting the findings reported in this article are available from the corresponding author upon reasonable request.
